# Muscle Fiber Characteristics and Transcriptome Analysis in Slow- and Fast-Growing *Megalobrama amblycephala*

**DOI:** 10.3390/genes15020179

**Published:** 2024-01-29

**Authors:** Xue Zou, Qi Liu, Qianqian Guan, Ming Zhao, Xin Zhu, Yaxiong Pan, Lusha Liu, Zexia Gao

**Affiliations:** 1 Key Lab of Agricultural Animal Genetics, Breeding and Reproduction of Ministry of Education, Key Lab of Freshwater Animal Breeding, Ministry of Agriculture, College of Fisheries, Huazhong Agricultural University, Wuhan 430070, China; zousnow@webmail.hzau.edu.cn (X.Z.); qiliu@webmail.hzau.edu.cn (Q.L.); guanqianqian@webmail.hzau.edu.cn (Q.G.); aam1207@webmail.hzau.edu.cn (M.Z.); gaozx@mail.hzau.edu.cn (Z.G.); 2 Department of Bioengineering and Environmental Science, Changsha University, Changsha 410003, China; xinzhu1219@163.com (X.Z.);; 3 Hubei Hongshan Laboratory, Wuhan 430070, China; 4 Engineering Technology Research Center for Fish Breeding and Culture in Hubei Province, Wuhan 430070, China

**Keywords:** *Megalobrama amblycephala*, skeletal muscle, growth difference, histological analysis, transcriptome analysis, hyperplasia, hypertrophy

## Abstract

Growth is an important trait in aquaculture that is influenced by various factors, among which genetic regulation plays a crucial role. *Megalobrama amblycephala*, one of the most important freshwater species in China, exhibits wide variations in body mass among individuals of the same age within the same pool. But the molecular mechanisms underlying wide variation in body mass remain unclear. Here, we performed muscle histological and transcriptome analysis of muscle tissues from Fast-Growing (FG) and Slow-Growing (SG) *M. amblycephala* at the age of 4 months old (4 mo) and 10 months old (10 mo) to elucidate its muscle development and growth mechanism. The muscle histological analysis showed smaller diameter and higher total number of muscle fibers in FG compared to SG at 4 mo, while larger diameter and total number of muscle fibers were detected in FG at 10 mo. The transcriptome analysis of muscle tissue detected 1171 differentially expressed genes (DEGs) between FG and SG at 4 mo, and 718 DEGs between FG and SG at 10 mo. Furthermore, 44 DEGs were consistently up-regulated in FG at both 4 mo and 10 mo. Up-regulated DEGs in FG at 4 mo were mainly enriched in the pathways related to cell proliferation, while down-regulated DEGs were significantly enriched in cell fusion and muscle contraction. Up-regulated DEGs in FG at 10 mo were mainly enriched in the pathways related to cell proliferation and protein synthesis. Therefore, these results provide novel insights into the molecular mechanism of *M. amblycephala* muscle growth at different stages, and will be of great guiding significance to promote the fast growth of *M. amblycephala*.

## 1. Introduction

Growth is one of the most important traits in aquaculture and fish growth is defined by a gradual increase in quantitative traits over time, such as body length, weight, and height [1]. The growth traits of fish are often regulated by multiple factors, including internal and external factors, among which genetic regulation plays a crucial role in growth, development, and metabolism [2,3]. Consequently, different individuals from the full-sib family often show wide variation in growth traits at the same age, which limits the development of aquaculture [4]. With the advancement of molecular genetics in fish populations, a better understanding of the molecular mechanism underlying growth traits is expected, and it can be utilized in marker-assisted selective breeding programs to promote rapid genetic improvement in fish growth traits [5]. Skeletal muscle constitutes approximately 50–70% of the total fish body mass, and it is the largest tissue in the fish body [6,7]. The growth and development of skeletal muscle significantly influence the overall growth of the fish, while muscle growth is mainly involved in hyperplasia (increase in muscle cell number) and hypertrophy (increase in muscle cell size) [8]. Therefore, understanding the differential expression of genes and regulative pathways in muscles is helpful for exploring the growth mechanisms of fish.

Several studies have investigated the genetic and molecular factors influencing the growth and development of various fish species through RNA-seq analysis of muscle tissues. RNA-seq analysis of Fast-Growing (FG) and Slow-Growing (SG) specimens of Hulong grouper (Hyb), *Ctenopharyngodon idella*, *Micropterus salmoides*, and *Scophthalmus maximus* found that glycolysis is an important pathway for the fast growth of fish muscles [9,10,11,12]. Another study on FG and SG *Spinibarbus denticulatus denticulatus* at different growth stages (90, 150, and 300 days after hatch) indicated that the growth hormone–insulin-like growth factor (GH/IGF) axis and adenosine monophosphate-activated protein kinase (AMPK) signal pathway may influence early growth differences, while the glycolytic pathway enriched in FG in the late growth stage [13]. In rice flower carp, the ubiquitin–proteasome pathway, protein synthesis-related genes, and muscle contraction activity genes were found to affect the growth rate, while the muscle histological analysis showed that the muscle growth in rice flower carp mainly depends on the hypertrophic growth of muscle fibers [4]. Zhang et al. (2020) found significant differences in the expression of some genes involved in growth- and development-related metabolic pathways between FG and SG *Mylopharyngodon piceus* [14]. Furthermore, the muscle growth of *Oncorhynchus mykiss* is influenced by genes related to blood and energy production, as well as immune function [15]. Therefore, RNA-seq is helpful to identify the candidate genes involved in fish growth, and the molecular regulatory mechanisms of growth in different fishes may be different. *M. amblycephala*, also known as blunt snout bream, is one of the most popular freshwater species in China. Since the 1960s, it has been recognized as a primary species in the polyculture system of Chinese freshwater fish due to advantages including excellent flesh quality, high nutritional and economic value, rapid growth, and affordability [16]. Liu et al. analyzed the complete transcriptome of the skeletal muscle of *M. amblycephala* with different growth rates, and found that two ceRNA regulatory networks associated with the phosphatidylinositol 3-kinase/protein kinase B (PI3K/AKT) signaling pathway and the apoptosis signaling pathway may play a crucial role in the growth and development of *M. amblycephala* [17]. But accurate and systematic understanding of the morphological characteristics of muscle and the molecular genetic basis for muscle growth and development at different growth stages has rarely been investigated in *M. amblycephala*. To elucidate the wide variation in body mass of *M. amblycephala* during development, integrated studies involving muscle histological studies and transcriptome analysis of FG and SG at different growth stages were conducted to identify the important candidate genes and pathways related to muscle growth and development in *M. amblycephala*. These results will provide a theoretical basis for disclosing the molecular mechanism of muscle growth and improve the production performance of *M. amblycephala*.

## 2. Materials and Methods

### 2.1. Ethics Statement

Animal experiments in this study were approved by the Animal Experimental Ethical Inspection of the Laboratory Animal Center, Huazhong Agricultural University, Wuhan, China (HZAUDO-2016-005, 26 October 2016), and all experiments were performed according to relevant guidelines and regulations. The dissection experiments were conducted using tricaine methanesulfonate (MS-222) (Sigma, Saint Louis, MO, USA; 100 mg/L) anesthesia to reduce the distress of the fish.

### 2.2. Experimental Animals

The *M. amblycephala* used in this study were bred in May 2022 and reared in the pond at Ezhou breeding base of Huazhong Agricultural University (HZAU). All the fish were fed under the same conditions in the same pool. We randomly collected *M. amblycephala* at 4 months (4 mo) and 10 months (10 mo) and selected the top 15% in terms of body weight which were assigned to the Fast-Growing (FG) group; the bottom 15% were assigned to the Slow-Growing (SG) group.

### 2.3. Date Measurement and Tissue Sampling

Growth traits, including full length, body length, and body weight, were measured to evaluate growth performance. After the experimental fish was anesthetized by MS-222 (100 mg/L), two pieces of white muscle tissue were taken from the muscle below the starting position of the dorsal fin and above the horizontal myoseptum (epaxial muscle). One part was frozen in liquid nitrogen and stored at −80 °C until RNA extraction, and the other part was stored in environmentally friendly GD solution (G1111, Servicebio, Wuhan, China) until histological processing. Three individuals of each group were used for histology analysis, RNA-seq, and gene expression analysis.

### 2.4. Muscle Histological Studies

To compare the differences in the diameter, area, and number of muscle fibers of *M. amblycephala* between FG and SG, we selected 6 muscle tissues (3 muscle tissues from each of FG and SG) for muscle histological analysis at 4 mo and 10 mo. Muscle samples were fixed in environmentally friendly GD solution for at least 24 h at room temperature, then embedded into paraffin wax and sectioned to 5 μm. The slides were then stained with hematoxylin–eosin (H&E) following routine protocols. Then, the slides were scanned digitally with the Pannoramic MIDI scanner (3DHISTECH Company, Budapest, Hungary). The CaseViewer software (version 2.4) was used to view the slices. At least three regions were randomly selected from each slice for analysis. The diameter and area of muscle fibers were measured and the total number of muscle fibers in the slide was counted by Image J software (version 1.53t). The diameter of the muscle fiber is defined as d = (major axis + minor axis)/2, and the area of the muscle fiber is defined as s = the total area of muscle fiber in a certain area/the number of muscle fibers.

### 2.5. RNA Extraction, Library Construction, and Sequencing

The total RNA was extracted from muscle tissues using RNAiso Plus (TaKaRa, Dalian, China). Following genomic DNA elimination, the RNA quality and integrity were evaluated and high-quality mRNA from FG and SG were used to construct libraries. The mRNA in the sample was enriched using magnetic beads with oligo (dT) and fragmented into short segments. The synthesis of the first strand cDNA utilized a random hexamer primer and M-MuLV Reverse Transcriptase (RNase H-), followed by the subsequent performance of second strand cDNA synthesis using DNA Polymerase I and RNase H. After 3′-end repair, ligation of a poly(A)-tail to the sequencing linker, and selection of the fragment sizes, the double-stranded cDNA was purified and PCR amplified. Then, the PCR products were purified using the AMPureXP system (Beckman Coulter, Beverly, MA, USA) for sequencing. Six cDNA libraries of 4 mo (FG and SG) and 10 mo (FG and SG) were sequenced using the Illumina sequencing platform (Illumina NovaSeq 6000) (Illumina, San Diego, CA, USA) by BioNovoGene Co. (Suzhou, China) and Novogene Bioinformatics Technology Co. (Beijing, China), respectively.

### 2.6. Quality Control and Comparative Analysis

The raw data (raw reads) in fastq format underwent initial processing using the fastp software (version 0.19.7). In this step, clean data (clean reads) were obtained by removing reads containing adapters, reads containing ploy-N (N represents reads for which base information cannot be determined), and low-quality reads from the raw data. The Q20, Q30, and GC-content of the clean data were calculated. All subsequent analyses were conducted based on the high-quality clean data. The index of the reference genome was constructed using Hisat2 v2.0.5, and the paired-end clean reads were aligned to *M. amblycephala* reference genome (ASM1881202v) using the same software version. The quantification of gene expression levels was performed as follows: the gene expression levels were estimated using the fragments per kilobase of transcript per million mapped fragments (FPKM) method [18].

The RNA-seq data utilized in this study have been deposited in the NCBI short read archive (SRA) and can be accessed under BioProject PRJNA1048634 and PRJNA104905.

### 2.7. Analysis of DEGs

The analysis of differential expression between FG and SG was conducted using the DESeq 2R package (version 1.20.0) with the readcount. The *p*-*adjust* obtained were adjusted using Benjamini and Hochberg’s approach to control the false discovery rate. *p*-*adjust* < 0.05 and |log2Fold-change| > 1 as the significance threshold were utilized to identify significant differences in transcriptional expression. Differentially expressed genes (DEGs) that exhibited overlapping up-regulation and down-regulation in FG at both 4 mo and 10 mo were identified by Novokin cloud platform (https://magic.novogene.com/customer/main#/homeNew) (accessed on 9 August 2023). DEGs were further annotated using GO and KEGG pathway analyses. A heatmap was plotted by bioinformatics (https://www.bioinformatics.com.cn) (accessed on 30 August 2023).

### 2.8. Real-Time PCR Confirmation of Illumina Sequencing Data

The transcript levels of 12 DEGs were validated using real-time quantitative reverse transcription PCR (qRT-PCR). The primers used for quantification in the study were designed using Primer-BLAST on the NCBI website (https://www.ncbi.nlm.nih.gov/tools/primer-blast/) (accessed on 3 November 2023) (Table 1). The amplification efficiency was determined using the gradient dilution method. The initial CT value was utilized for calculating the primer amplification efficiency and R^2^, following the formula E = (10^−1/k^ − 1) × 100%, with the slope being derived from the linear regression equation in Excel (version 2016) (Table S1). In the study, β-actin was used as the housekeeping gene. The number of replications for each sample was three. The qPCR was conducted on QuantStudio 6 Flex Real-Time PCR System (Life Technologies, Carlsbad, CA, USA) in a total volume of 20 μL with 10 μL of HieffTM qPCR SYBR^®^ Green Master Mix (Yeasen, Shanghai, China), 0.5 μL of each primer, and 9 μL of the diluted cDNA. The cycling parameters were 95 °C for 5 min, followed by 40 cycles of 95 °C for 10 s, 62 °C for 20 s, and 72 °C for 20 s. Melting curve analyses were performed following the amplifications.

### 2.9. Statistical Analysis

All data were presented as mean ± standard deviation. Excel was used for data statistics and sorting, and then GraphPad Prism 8.0 was used for statistical analysis and mapping. Significant differences were analyzed via t tests and indicated by an asterisk and lowercase letter at *p* < 0.05.

## 3. Results

### 3.1. Growth Performance of FG and SG M. amblycephala

**Figure 1:**
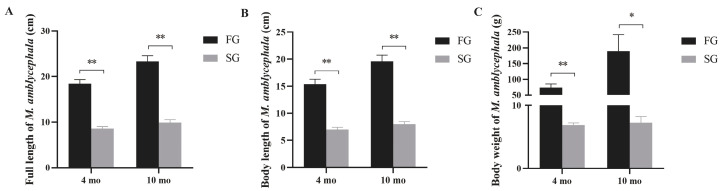
Comparison of total length (**A**), body length (**B**), and weight (**C**) between FG and SG at different growth stages. Asterisks indicate significant differences between FG and SG. * *p* < 0.05, ** *p* < 0.01.

The top 15% of FG and SG samples at different growth stages were collected for growth performance analysis. The statistical results showed that FG individuals had significantly higher total length, body length, and body weight than SG individuals at 4 mo and 10 mo (*p* < 0.01) (Figure 1). Especially in terms of body weight, the average body weights of FG and SG were 67.26 ± 8.012 and 6.87 ± 0.35 at 4 mo, and 157 ± 25.46 and 7.25 ± 0.96 at 10 mo, respectively. At 4 mo and 10 mo, the weight of FG individuals increased by about 10 times and 22 times compared with SG, respectively.

### 3.2. Muscle Histological Analysis

**Figure 2:**
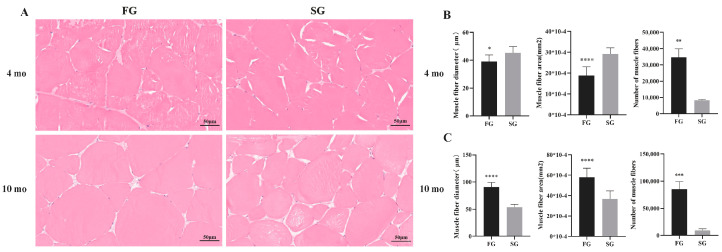
(**A**) The muscle fibers of the FG and SG at different growth stages were observed in 300× microscope, scale bar: 50 μm. (**B**) Histogram of muscle fiber diameter, area, and number in FG and SG of *M. amblycephala* at 4 mo. (**C**) Histogram of muscle fiber diameter, area, and number in FG and SG of *M. amblycephala* at 10 mo. * *p* < 0.05, ** *p* < 0.01,*** *p* < 0.001, **** *p* < 0.0001.

H&E staining section showed the anatomical structure of muscles, and the statistical results of muscle fiber diameter, area, and number in different groups are shown in Figure 2. There were significant differences in the muscle diameter, fiber area, and total number of muscle fibers in the FG and SG. At 4 mo, the diameter and area of muscle fibers in FG were significantly smaller than those in SG (*p* < 0.05). However, the total number of muscle fibers in FG was significantly higher than in SG (*p* < 0.005). At 10 mo, the diameter, area, and total number of muscle fibers in FG were significantly larger than those in SG (*p* < 0.001).

### 3.3. Analysis of DEGs between FG and SG at Different Growth Stages

Clean reads were prepared by eliminating adapter fragments and low-quality bases from the raw data. As shown in Table S2, the raw reads, raw bases, clean reads, clean bases, and Q30 were recorded for 12 libraries. For all libraries, high-quality (Q30 ≥ 93%) sequencing data will be used for subsequent analyses.

**Figure 3:**
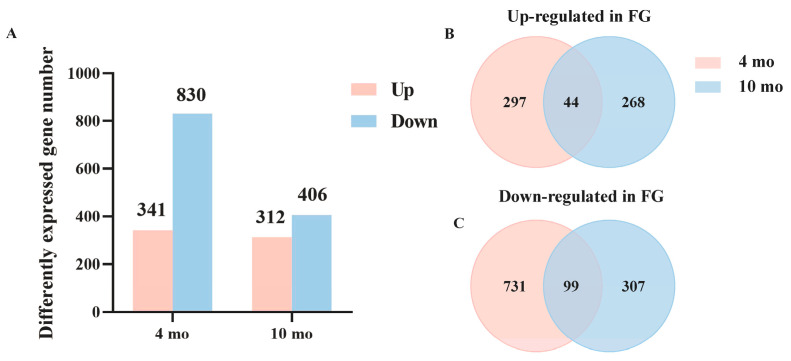
Analysis of DEGs at different growth stages. (**A**) Numbers of up- or down-regulated genes at 4 mo and 10 mo. (**B**) DEGs’ Venn diagram was up-regulated in FG at 4 mo and 10 mo. (**C**) DEGs’ Venn diagram was down-regulated in FG at at 4 mo and 10 mo.

Genes were considered as DEGs if they showed a *p*-*adjust* < 0.05 and |log2Fold-change| > 1. Using SG as the control group, DEGs analysis showed that there were 1171 DEGs between FG and SG in 4 mo, of which 341 genes were up-regulated in FG and 830 genes were down-regulated in FG. Meanwhile, there were 718 DEGs between FG and SG at 10 mo, including 312 up-regulated genes and 406 down-regulated genes in FG compared to SG (Figure 3A). Then, we compared FG up-regulated genes that overlapped in two developmental stages. As shown by Venn diagram, 44 genes were significantly up-regulated in FG at both 4 mo and 10 mo (Figure 3B), and 99 genes were significantly down-regulated in FG at these two development stages (Figure 3C).

### 3.4. Enrichment Analysis of GO and KEGG Pathways Based on DEGs at 4 mo

The GO enrichment was used to classify the DEGs into three categories: biological process, molecular function, and cell composition. In total, 853 GO terms were significantly enriched at 4 mo (*p*-*adjust* < 0.05). The top one GO classification of biological processes was ATP metabolic process (GO:0046034), while the top one GO classification of molecular function was NADH dehydrogenase (ubiquinone) activity (GO:0008137). The top two GO classifications of cellular components were myofibril (GO:0030016), and sarcomere (GO:0030017) (Table S3). In addition, GO analysis of down-regulated DEGs showed that significant changes in ATP metabolic process (biological process), troponin C binding (molecular function), and myofibril (cell component). In the category of cell components, there were many terms related to skeletal muscle structure (myofibril assembly, myofibril, etc.) and myoblast fusion (myoblast fusion, cell–cell fusion, regulation of myoblast fusion, etc.) (Table S4).

**Figure 4:**
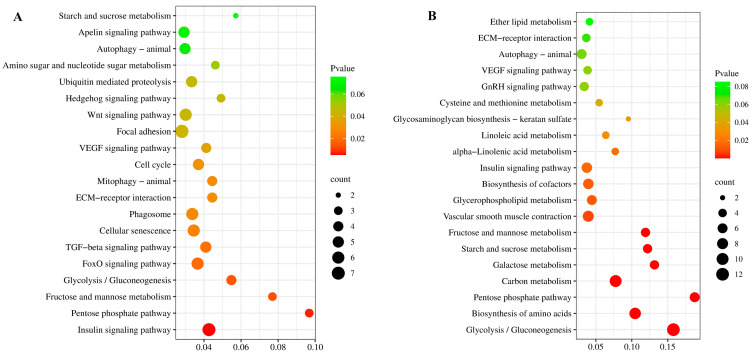
Scatterplots of significantly enriched KEGG pathways by up-regulated genes in FG vs. SG at 4 mo (**A**) and 10 mo (**B**).

For KEGG enrichment analysis, total DEGs were significantly enriched to 26 pathways (*p*-value < 0.05) (Table S5). Further KEGG analysis of up-regulated and down-regulated DEGs showed that the up-regulated DEGs were mainly enriched in the insulin signaling pathway (Figure 4A), while the down-regulated DEGs were mainly enriched in cardiac muscle contraction (Figure S1). Furthermore, some pathways related to cell proliferation were significantly enriched in the up-regulated pathway, such as the “Insulin signaling pathway” (seven DEGs), “FoxO signaling pathway” (six DEGs), “Cell Cycle” (five DEGs),“TGF-β signaling pathway” (five DEGs), “ECM-receptor interaction” (four DEGs), “Wnt signaling pathway” (six DEGs), “Hedgehog signaling pathway” (three DEGs) (Figure 4A).

### 3.5. Enrichment Analysis of GO and KEGG Pathways Based on DEGs at 10 mo

At 10 mo, a total of 88 GO terms were significantly enriched (*p*-*adjust* < 0.05). The top one GO classification of biological processes was peptide biosynthetic process (GO:0043043). The top one GO classification of molecular function was structural molecule activity (GO:0005198). The top one GO classification of cellular components was actin cytoskeleton (GO:0015629) (Table S6).

KEGG enrichment analysis showed that the total DEGs significantly enriched 19 pathways (*p*-value < 0.05) (Table S7). Further KEGG analysis of up-regulated and down-regulated DEGs showed that the up-regulated DEGs were mainly enriched in glycolysis/gluconeogenesis (Figure 4B), while the down-regulated DEGs were mainly enriched in ribosome (Figure S2). Through the significant pathway enrichment analysis, some pathways related to cell proliferation were significantly enriched in the up-regulated pathway, such as “Insulin signaling pathway” (seven DEGs) and some pathways related to protein synthesis were identified, such as “Biosynthesis of amino acids” (nine DEGs) and “Carbon metabolism” (ten DEGs) (Figure 4B).

### 3.6. Synergistic Regulation of Gene Categories Related to Muscle Growth in M. amblycephala at Different Growth Stages

**Figure 5:**
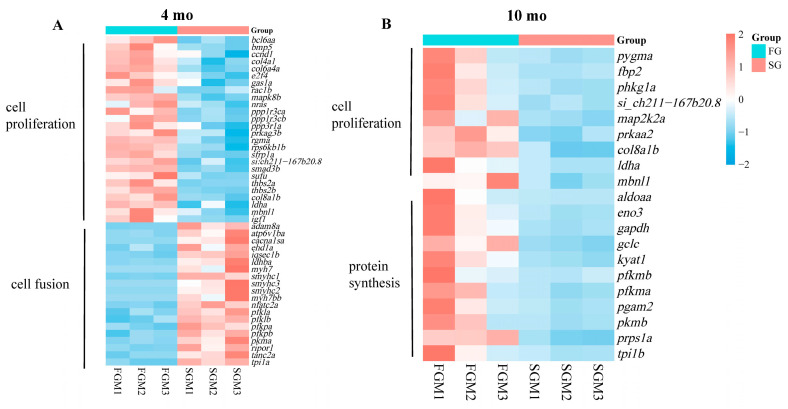
Heatmaps showing coordinated regulation of DEGs in different cell processes between FG and SG. (**A**) Thermograms of 20 DEGs related to cell proliferation and 22 DEGs related to cell fusion at 4 mo. (**B**) Thermograms of 7 DEGs related to cell proliferation and 11 DEGs related to protein synthesis at 10 mo.

The genes enriched in the pathways/processes related to muscle growth were further compared and integrated into the 4 mo and 10 mo heatmaps, respectively. Up-regulated DEGs in FG at 4 mo were mainly enriched in the pathways related to cell proliferation, while down-regulated DEGs were significantly enriched in cell fusion and muscle contraction (Figure 5A). However, up-regulated DEGs in FG at 10 mo were mainly enriched in the pathways related to cell proliferation and protein synthesis (Figure 5B).

### 3.7. Comparative Analysis of DEGs in 4 mo and 10 mo M. amblycephala

**Figure 6:**
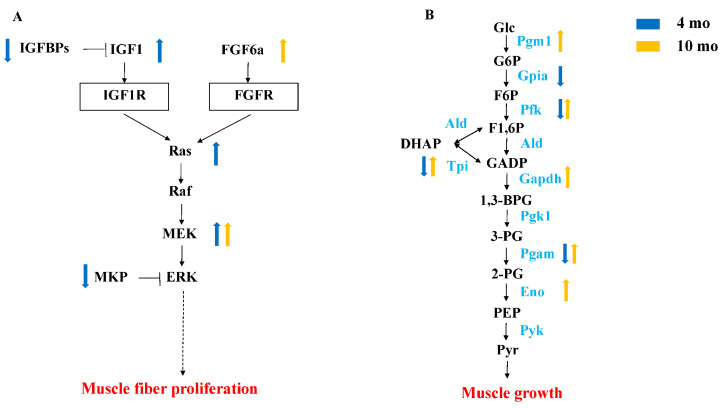
Comparative analysis of DEGs at different growth stages. (**A**) Schematic diagram of MAPK signaling pathway. (**B**) Schematic diagram of glycolytic pathway. The blue arrow represents DEGs in FG vs. SG at 4 mo, and the yellow arrow represents DEGs in FG vs. SG at 10 mo.

Forty-four genes were significantly up-regulated and expressed in FG at both 4 mo and 10 mo, including *col8a1b*, *ldha*, and *mbnl1* (Table S8). In order to understand the pathways involved in growth differences, we focused on the two growth-related pathways enriched by overlapped DEGs. The growth-related pathways enriched by DEGs in the muscle were the insulin signaling pathway and glycolysis (Figure 6). Through the insulin signaling pathway, *igf1*, *NRAS proto*-*oncogene*, *GTPase* (*nras*), and *map2k2a* were significantly up-regulated at 4 mo, and *fibroblast growth factor 6a* (*fgf6a*) and *map2k2a* were also significantly up-regulated at 10 mo. In the glycolytic pathway, *glucose*-*6*-*phosphate isomerase a* (*gpia*), *phosphofructokinase*, *liver a* (*pfkla*), *phosphofructokinase*, *liver b* (*pfklb*), *phosphofructokinase*, *platelet a* (*pfkpa*), *phosphofructokinase*, *platelet b* (*pfkpb*), *triosephosphate isomerase 1a* (*tpi1a*), and *phosphoglycerate mutase 1a* (*pgam1a*) were highly expressed in SG at 4 mo, and *phosphoglucomutase 1* (*pgm1*), *pfkma*, *tpi1b*, *gapdh*, *pgam2*, and *enolase 3* (*eno3*) were highly expressed in FG at 10 mo.

### 3.8. Verification of RNA-Seq Data by qPCR

**Figure 7:**
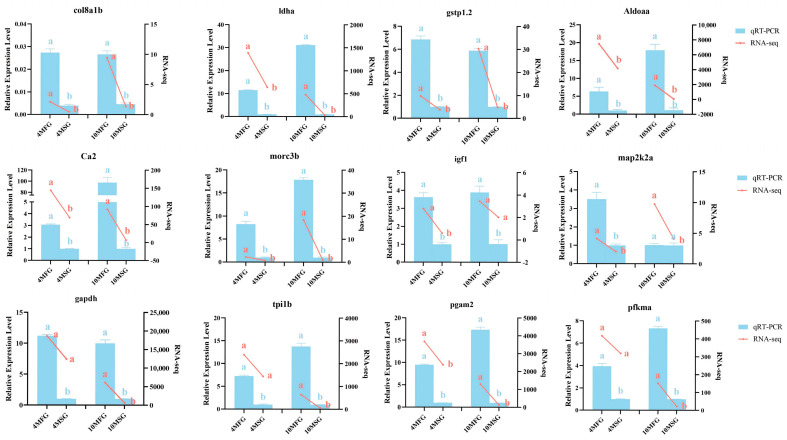
RT-qPCR verification of differentially expressed DEGs. The right vertical axis indicates RNA-seq expression level. The left vertical axis indicates RT-qPCR relative expression level. Different lowercase letters represent significant differences (*p* < 0.05).

To confirm the reliability of the RNA-seq data, six genes (*col8a1b*, *ldha*, *gstp1.2*, *Aldoaa*, *ca2*, *morc3b*) up-regulated at 4 mo and 10 mo, two genes (*igf1*, *map2k2a*) involved in the MAPK signaling pathway, and four genes (*gapdh*, *tpi1b*, *pgam2*, *pfkma*) from glycolytic pathway were selected for qPCR analysis. The analysis of amplification efficiency and correlation coefficient revealed that the amplification efficiency of all genes ranged between 92% and 108%, except for igf1, which exhibited a higher amplification efficiency of 115%. Furthermore, the linear correlation coefficient R^2^ for all genes exceeded 0.99, which indicated that the above primers had strong specificity and good amplification efficiency. qRT-PCR results showed that the expression patterns of these genes were highly consistent with the sequencing results (Figure 7).

## 4. Discussion

Growth is one of the most important economic traits for fish breeding. The skeletal muscle, contributing about 60% to the fish body weight, significantly influences fish growth traits through its development and growth. Muscle growth primarily involves two processes: hyperplasia, characterized by the formation of new fibers, and hypertrophy, marked by an increase in muscle fiber diameter [8]. But genes and genetic pathways regulating muscle growth in teleost are not clear. In this study, histology analysis and transcriptomes of FG and SG individuals at 4 mo and 10 mo were analyzed to screen for differentially expressed genes and pathways potentially influencing muscle growth and development of *M. amblycephala*.

Our study is the first report of the muscle histology analysis in *M. amblycephala* between FG and SG. At 4 mo, smaller diameter and area of muscle fibers and higher total number of muscle fibers in FG were detected compared to SG. These findings suggest that hyperplasia plays a more significant role in the early growth of *M. amblycephala*. At this stage, proliferation of muscle cells results in the fast growth of *M. amblycephala*. At 10 mo, the diameter, area, and total number of muscle fibers in FG were significantly larger than those in SG, indicating both the augmentation of muscle cell number and the expansion of cell size becoming crucial factors contributing to its overall growth in *M. amblycephala* late growth. Histological analysis revealed that fast growth relied on both hyperplasia and hypertrophy at the late growth stage (10 mo). These results suggested that muscle fiber proliferation is more crucial in the early growth stage of *M. amblycephala*, while as *M. amblycephala* develops, both the augmentation of muscle cell number and cell size become vital for its growth. In 20-month-old common carp, the hypertrophy of muscle fibers was an important reason for the difference in growth [4]. Compared with two-year-old *Coregonus maraena*, the hypertrophy of muscle fibers is an important reason for the rapid growth of one-year-old *Oncorhynchus mykiss* [19]. These results indicate that hypertrophic growth of muscle plays an important role in the growth of skeletal muscle in the later development stage in many species, and the role of hyperplasia and hypertrophy in muscle growth depends on the developmental stage and species.

At the early growth stage (4 mo), 341 genes were up-regulated in FG compared to SG. Of these up-regulated genes, sixteen up-regulated KEGG pathways were significantly enriched, including the insulin signaling pathway, glycolysis/gluconeogenesis, and FoxO signaling pathway. These pathways related to muscle development have also been enriched in FG common carp [4]. The insulin signaling pathway coordinates systemic growth, development, and peripheral and central nutritional homeostasis. In vertebrates, the insulin signaling pathway controls the growth of cells and tissues [20]. The insulin signaling pathway also exhibited significant enrichment in Fast-Growing chickens and *Mylopharyngodon piceus*, suggesting its pivotal role in the observed growth discrepancy [14,21]. In addition, the TGF-β signaling pathway, extracellular matrix (ECM)-receptor interaction, Wnt signaling pathway, Hedgehog signaling pathway, and cell cycle have been enriched in FG. Substantial evidence has verified the significant involvement of the TGF-β pathway in the proliferation, differentiation, and migration of muscle cells [22], thus suggesting its implication in the growth variation of *M. amblycephala*. Moreover, the extracellular matrix (ECM) is crucial for various cellular responses, including transcription, inflammation, migration, proliferation, and differentiation [23]. Studies have shown that ECM–receptor interaction plays a key role in chicken muscle growth and development [24,25]. Wnt signaling has been shown to play a role in the development of muscle during embryogenesis [26,27]. Teixeira et al. found that the Sonic Hedgehog signaling pathway was involved in the proliferation of chick embryonic myoblasts and the terminal differentiation of muscle fibers [28]. These findings provide a great deal of evidence to support their role in muscle development of *M. amblycephala* FG. During the late growth stage (10 mo), the insulin signaling pathway is also significantly enriched in FG. This indicates that the insulin signaling pathway plays an important role in the muscle growth of *M. amblycephala* at different development stages. Furthermore, a significant increase in the expression of DEGs related to protein synthesis has been identified, such as *aldoaa*, *tpi1b*, *pfkma*, etc. The hypertrophy and growth of muscle fibers depend not only on the fusion of myoblast, but also the protein deposition in mature muscle fibers [29]. Additionally, we found that two pathways related to protein synthesis (biosynthesis of amino acids and carbon metabolism) were significantly enriched in FG at 10 mo. Through a combination of histology and transcriptome analysis, it was found that muscle growth is dependent on both hyperplasia and hypertrophy at the late growth stage (10 mo). This also indicates that increased protein deposition leading to hypertrophic growth of muscle fibers contributes to the growth differences in *M. amblycephala* at 10 mo, which has been found in common carp [4].

Comparative analysis of DEGs between 4 mos and 10 mos showed that the expression levels of 44 genes were higher in FG at both 4 mo and 10 mo, such as *col8a1b* and *muscleblind-like splicing regulator 1* (*mbnl1*). Several studies have demonstrated the ability of COL8A1 to enhance cell proliferation [30,31]. And Song et al. (2020) discovered that MBNL1 can promote the proliferation of skeletal muscle satellite cells by inhibiting autophagy through the mammalian target of rapamycin (mTOR) pathway [32]. Together with the histology results, these 44 genes will be the candidate genes most important for muscle cell proliferation.

Interestingly, some up-regulated pathways had been enriched at two development stages, such as the insulin signaling pathway and glycolysis/gluconeogenesis. In conjunction with histological results, this indicates that the insulin pathway regulates hyperplasia in *M. amblycephala*. The insulin pathway plays an important role in proliferation. Research has demonstrated that insulin’s pro-proliferative effect is associated with the phosphorylation activation of c-Jun N-terminal kinase (JNK) and extracellular signal-related kinase (ERK 1/2), with JNK playing a predominant role in mediating insulin-induced cell proliferation [33]. At 4 mo, the insulin signaling pathway was significantly enriched, and related genes such as Ras (*nras*) and JNK (*mapk8b*) were significantly up-regulated in FG. The IGF system, extensively studied in teleost fishes and mammals for its role in growth [34,35,36,37], is composed of IGF (IGF1 and IGF2), IGF receptors (IGF1R and IGF2R), and IGF-binding proteins (IGFBP) [38]. Within the circulatory system, IGFBP binds to IGF, facilitating its transportation and increasing its half-life by preventing proteolytic degradation [39]. Research indicates that IGFBP6 inhibits the actions of IGF2 and IGF1. Overexpression of *igfbp6* (*igfbp6a/b*) in zebrafish has been associated with various growth impairments, impacting body size and delaying normal development in embryos [40]. The present study observed that the high expression of *igf1* in the FG of *M. amblycephala* at 4 mo activated the insulin signaling downstream pathway (MAPK signaling pathway) to facilitate the proliferation of muscle cells. Furthermore, the reduced expression of *igfbp6b* in FG muscle suggested its potential role as a negative indicator of *M. amblycephala* rapid growth. Fibroblast growth factor 6 (FGF6), a member of the FGF family, is known for its specific expression during skeletal muscle development and potential involvement in muscle maintenance and regeneration [41]. Previous studies in adult trout (100 to 500 g) have demonstrated that the expression of *FGF6* is linked to the continuous proliferation of white muscle [41]. Furthermore, Xu et al. discovered that *FGF6* promotes myofiber proliferation in grass carp across various growth stages and tissues through bioinformatics analysis and expression profiling [42]. Additionally, Cai et al. established that FGF6 stimulates myoblast proliferation and differentiation by activating ERK-dependent pathways via the MAPK signaling pathway [43]. Studies have shown that the proliferation-promoting effect of insulin has been demonstrated to be primarily mediated by the Akt cascade and activation of the MAPK pathway [21,44]. This study also found high expression of *FGF6a* in FG at 10 mo, accompanied by heightened expression of its downstream gene *map2k2a*, indicating potential activation of the ERK-dependent pathway. These findings suggest that *FGF6a* may facilitate sustained myofiber proliferation through the insulin signaling pathway during the adult stage of *M. amblycephala*.

It was reported that glycolysis was involved in the development of fish muscles. The muscle tissue of the Fast-Growing hybrid grouper (Hulong grouper) exhibited significantly higher expression levels of almost all glycolytic genes compared to its parental species [9]. Similarly, heightened expression of glycolytic enzyme-related genes in the Fast-Growing *Ctenopharyngodon idella* signifies increased metabolic activity in the white muscle, aligning with the heightened energy demand of rapidly growing animals [10]. Furthermore, significant enrichment of the glycolytic pathway was observed in the rapidly growing *Micropterus salmoides* and *Scophthalmus maximus* [11,12]. Mutations in glycolytic genes such as Pfkm, Pgam, and Pyk can lead to various muscle-related disease symptoms. Tixier et al. identified six genes involved in glycolysis or pyruvate metabolism (*Pfk*, *Tpi*, *Gapdh*, *Pgk*, *Pyk*, and *Impl3*) in zebrafish and demonstrated that their reduced expression resulted in decreased muscle fiber size and the presence of unfused myoblasts [45,46] Notably, at 4 mo, genes encoding key glycolytic enzymes such as *gpia*, *pfkla*, *pfklb*, *pfkpa*, *pfkpb*, *tpi1a*, and *pgam1a* were highly expressed in SG, whereas at 10 mo, genes encoding key glycolytic enzymes such as *pgm1*, *pfkma*, *tpi1b*, *gapdh*, *pgam2*, and *eno3* were highly expressed in FG. These findings emphasize the crucial function of these genes on myofiber hypertrophy in *M. amblycephala*. The expression of these genes may facilitate the hypertrophy of myofibers in *M. amblycephala*, thus leading to increased muscle fiber diameter.

## 5. Conclusions

In conclusion, the molecular mechanisms underlying muscle development and growth in *M. amblycephala* were elucidated by comparing the transcriptome of FG and SG at different development stages. Based on the histological results, we found that the early growth stage (4 mo) of *M. amblycephala* mainly depended on the proliferation of muscle fibers, while the late growth stage (10 mo) mainly depended on the hypertrophy and proliferation of muscle fibers. Our transcriptome results suggest that the insulin signaling pathway and MAPK pathway are important reasons for muscle fiber proliferation of *M. amblycephala* at 4 mo and 10 mo. In addition, glycolysis and protein production promoted the hypertrophy and growth of muscle fibers to some extent.

## Figures and Tables

**Table 1:** Primers employed for gene expression analysis via quantitative real-time PCR.

Gene	Full Gene Name	Forward Primer	Reverse Primer
*col8a1b*	*collagen*, *type VIII*, *alpha 1b*	ACCCATTGTAGGAAAAGGTGAA	CCAGGTTTACCTCCAGGTCC
*ldha*	*lactate dehydrogenase A4*	GAGACTCCAGCGTTCCTGTG	CACCATCTTGTGGACGCTCT
*gstp1.2*	*glutathione S-transferase pi 1.2*	CCCGATCCTACTACACGATGGC	GATCACCCTTCATCCACTCATCA
*Aldoaa*	*aldolase a, fructose-bisphosphate, a*	GGAATGTCAAAGGTCATGCCG	GTGAGGCATCTTGACTCGTGTA
*ca2*	*carbonic anhydrase II*	ACGACAGCTCAACACTGACA	GAGCTCGGCTGGGTAACATT
*morc3b*	*MORC family CW-type zinc finger 3b*	TGTAGCCAGTCTGAGAGCGA	CCATTTGTGGTCGTGCGAAG
*Igf1*	*insulin-like growth factor 1*	ACATTGCCCGCATCTCATCC	CAGCGCATGGTACACTTAAAGA
*map2k2a*	*mitogen-activated protein kinase kinase 2a*	GGTCATGGCCAGGAAGCTTAT	CACTGTAGAAGGCGCCGTAA
*gapdh*	*glyceraldehyde-3-phosphate dehydrogenase*	GCCTTGAGAAACCTGCCAAG	CCCGTTGAAGTCAGTGGACA
*tpi1b*	*triosephosphate isomerase 1b*	AAAAACTGCATCGCCCCAAC	CCGGTGACAGACCCTCCATA
*pgam2*	*phosphoglycerate mutase 2* (*muscle*)	GACACCATTGCTCGTGCTCT	GCTGCATCTGACATGCTCTCTA
*pfkma*	*phosphofructokinase, muscle a*	CCCGGCGACAAAGAGTAACA	CAAAGCCATCATGAACCGCC
*β-Actin*	*actin, beta 2*	CGTGCTGTTTTCCCTTCCATT	CAATACCGTGCTCAAAGGATACTT

## Data Availability

The RNA-seq data utilized in this study have been deposited in the NCBI short read archive (SRA) database with accession number PRJNA1048634 and PRJNA104905.

## Supplementary Materials

The following supporting information can be downloaded at: https://www.mdpi.com/article/10.3390/genes15020179/s1, Figure S1. Scatterplots of significantly enriched KEGG pathways by down-regulated genes in FG vs. SG at 4 mo. Figure S2. Scatterplots of significantly enriched KEGG pathways by down-regulated genes in FG vs. SG at 10 mo. Table S1. Analysis of primer sequence and PCR amplification characteristics. Table S2. Summary of transcriptome data generated from *M. amblycephala* samples. Table S3. The GO enrichment results of total DEGs in *M. amblycephala* at 4 mo. Table S4. The GO enrichment results of down-regulated DEGs in *M. amblycephala* at 4 mo. Table S5. The KEGG enrichment results of total DEGs in *M. amblycephala* at 4 m. Table S6. The GO enrichment results of total DEGs in *M. amblycephala* at 10 mo. Table S7. The KEGG enrichment results of total DEGs in *M. amblycephala* at 10 mo. Table S8. Genes up-regulated in FG at both 4 mo and 10 mo.
