# Supplementary figures and images for: Muscle Fiber Characteristics and Transcriptome Analysis in Slow- and Fast-Growing Megalobrama amblycephala
Source: Genes (Basel). 2024 Jan 29;15(2):179. doi: 10.3390/genes15020179 (PMC10888202; doi:10.3390/genes15020179)

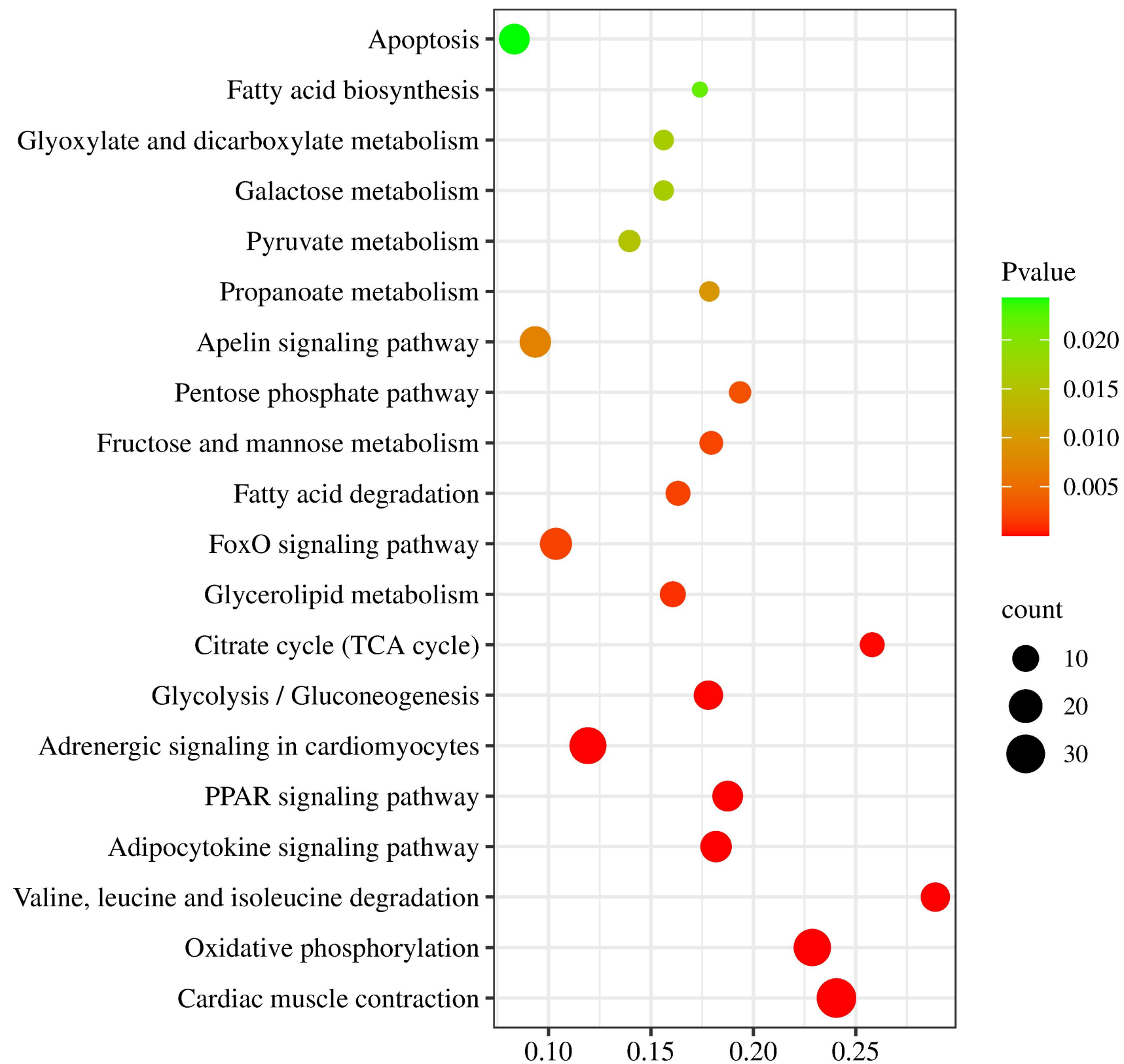

Supplement: Supplementary file 1 [file genes-15-00179-s001.zip › FigureS1.pdf]

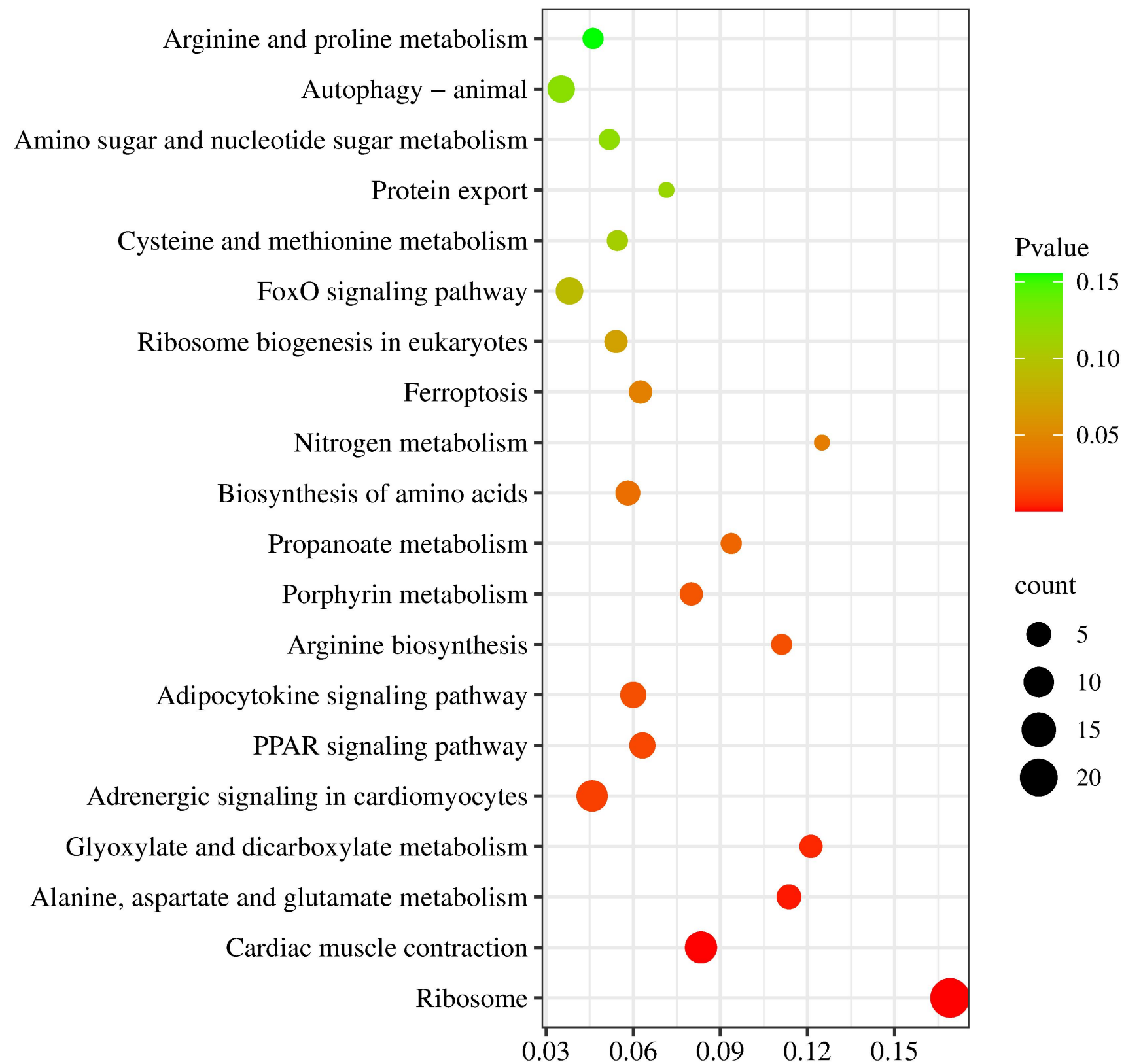

Supplement: Supplementary file 1 [file genes-15-00179-s001.zip › FigureS2.pdf]
